# Antenatal screening for HIV, hepatitis B and syphilis in the Netherlands is effective

**DOI:** 10.1186/1471-2334-11-185

**Published:** 2011-06-30

**Authors:** Eline LM Op de Coul, Susan Hahné, Yolanda WM van Weert, Petra Oomen, Colette Smit, Kitty PB van der Ploeg, Daan W Notermans, Kees Boer, Marianne AB van der Sande

**Affiliations:** 1 Centre for Infectious Disease Control, National Institute for Public Health and the Environment, Bilthoven, the Netherlands; 2 Stichting HIV Monitoring, Academic Medical Centre of the University of Amsterdam, the Netherlands; 3 TNO quality of life, department of Prevention and Healthcare, Leiden, the Netherlands; 4 Laboratory of Infectious Diseases and Perinatal Screening, National Institute for Public Health and the Environment, Bilthoven, the Netherlands; 5 Academic Medical Centre, department of gynaecology, University of Amsterdam, the Netherlands

## Abstract

**Background:**

A screening programme for pregnant women has been in place since the 1950s in the Netherlands. In 2004 universal HIV screening according to opting out was implemented. Here, we describe the evaluation of the effectiveness of antenatal screening in the Netherlands for 2006-2008 for HIV, hepatitis B virus (HBV) and syphilis in preventing mother-to-child transmission, by using various data sources.

**Methods:**

The results of antenatal screening (2006-2008) were compared with data from pregnant women and newborns from other data sources.

**Results:**

Each year, around 185,000 pregnant women were screened for HIV, HBV and syphilis. Refusal rates for the screening tests were low, and were highest (0.2%) for HIV. The estimated annual prevalence of HIV among pregnant women was 0.05%.

Prior to the introduction of screening, 5-10 children were born with HIV annually After the introduction of screening in 2004, only 4 children were born with HIV (an average of 1 per year). Two of these mothers had become pregnant prior to 2004; the third mother was HIV negative at screening and probably became infected after screening; the fourth mother's background was unknown. Congenital syphilis was diagnosed in fewer than 5 newborns annually and 5 children were infected with HBV. In 3 of these, the mothers were HBeAg positive (a marker for high infectivity). We estimated that 5-10 HIV, 50-75 HBV and 10 syphilis cases in newborns had been prevented annually as a result of screening.

**Conclusions:**

The screening programme was effective in detecting HIV, HBV and syphilis in pregnant women and in preventing transmission to the child. Since the introduction of the HIV screening the number of children born with HIV has fallen dramatically.

**Previous publication:**

[Translation from: 'Prenatale screening op hiv, hepatitis B en syphilis in Nederland effectief', published in 'The Dutch Journal of Medicine ' (NTVG, in Dutch)]

## Background

[Translation from: 'Prenatale screening op hiv, hepatitis B en syphilis in Nederland effectief', first published in 'The Dutch Journal of Medicine ' by Op de Coul EL, van Weert JW, Oomen PJ, Smit C, Hahné SJ, Notermans DW, Van der Sande MA: *Ned Tijdschr Geneeskd *2010, **154: **A2175 [1]

A screening programme for pregnant women has been in place since the 1950s in the Netherlands. Initially the programme exclusively consisted of determining the ABO blood group and detecting syphilis antibodies. In the 1970s, the Rhesus (D) factor was added. To this were added, the hepatitis B (HBV) carrier test in 1989, the irregular erythrocytes antibodies (IEA) test in 1998 and in 2004 the HIV antibody test.

The blood sample is collected during the first midwife appointment (preferably before the 13th week of the pregnancy) according to the opting-out principle, whereby all pregnant women undergo the test after having been provided with information, unless they explicitly state they do not wish to participate.

An intervention will take place following an abnormal result for one or more infectious diseases during and/or after the pregnancy in order to limit the risk of congenital (intra-uterine) and perinatal infections (around the pregnancy) to the child [2,3]. Virtually all pregnant women in the Netherlands participate in this infectious diseases screening programme [4,5].

We examined the effectiveness of HIV, HBV and syphilis screening in 2006-2008. We investigated how many pregnant women and newborns were infected with HIV, HBV, or syphilis and how many congenital/perinatal infections were prevented through timely interventions (immunisation of the child, e.g. HBV) or treatment of the mother (HIV, syphilis).

## Methods

Since 2005, the screening results from pregnant women have been collected in a national electronic database (Praeventis). Details on participation and screening results are collected by regional coordination programmes (RCP) by midwifes and regional laboratories. If the result of the first screening test is abnormal then other tests are carried out for confirmation. The HIV diagnosis is based on an ELISA, followed by an immunoblot or RNA test. HBsAg is determined for HBV followed by a confirmatory HBsAg test and tests for HBeAg (marker for high infectivity), anti-HBc and anti-Hbe (marker for low infectivity). Syphilis is determined using TPHA/TPPA (*Treponema pallidum *haemagglutination, *T. pallidum *particle agglutination) tests followed by a 'venereal disease research laboratory' (VDRL)-test and/or a fluorescent treponemal antibody (FTA) absorption test.

To determine the prevalence, all confirmed positive results were counted, regardless of the type test.

Prior to the impact evaluation, various process evaluations of the screening programme were conducted. These showed that registration of the confirmatory tests following an abnormal initial blood test was incomplete [4,6]. The results of these tests were not provided unambiguous by laboratories which meant that the definitive test result (positive or negative HIV, HBV or syphilis status) was often not clear. For this reason, the missing confirmatory results and conclusions from 2006-2008 were requested from laboratories. Of the 212 missing HIV confirmatory results, 44 (21%) were retrieved (17 positive and 27 negative). Of the 404 missing syphilis results, 74 were retrieved (18%; 48 positive, 26 negative) and of the 160 missing HBV results 47 (29%; 35 positive and 12 negative). Unfortunately, it was not possible to retrieve all definitive results. Therefore the prevalence of infectious diseases was estimated in three ways: (1) as minimum prevalence: the number of confirmed positive test results in pregnancies divided by the total number of pregnancies screened; (2) under the assumption that pregnant women with a positive result at the initial blood test, but no confirmation, were equally likely to have had a positive result as those pregnant women who have had confirmation; (3) under the assumption that all pregnant women without a confirmatory result were positive cases (maximum prevalence).

The effectiveness of screening for HIV, syphilis and HBV was calculated on the basis of the prevention of these infections in pregnant women and newborns. For this calculation Praeventis data were compared with other data sources (Dutch National Medical Register - Landelijke Medische Registratie (LMR), RIVM-LIS (Laboratory for Infectious Diseases and Perinatal Screening - Laboratorium voor Infectieziekten en Screening) and Osiris (compulsory notification of infectious diseases)). We compared the number of pregnant women screened, women refusing to be tested and results, and the demographic characteristics of the women with a positive test result, where available. For each disease, the contribution of these data sources was as follows.

### HIV

No distinction is made in Praeventis between newly diagnosed HIV and known HIV infected. Therefore, these data were obtained from the 'Stichting HIV Monitoring' (SHM), including HIV test results from newborns [7].

### Hepatitis B

The RIVM evaluates the HBV vaccination programme for children of HBsAg positive mothers [8,9]. Data from this evaluation were compared with the HBsAg and/or HBeAg positive results from pregnant women registered in Praeventis. An estimate of the number of known versus new HBV positive mothers was made based on a comparison of HBV positive pregnant women in Osiris (newly diagnosed) and Praeventis.

### Syphilis

Compulsory notification of syphilis came to an end in 1999 and since then there has been no nationwide registration system for monitoring of congenital syphilis (CS). Two alternative data sources were investigated in order to acquire an insight into the number of CS cases in the Netherlands. The RIVM performs IgM serodiagnostics in neonates and young children (<1 year) who are suspected of having CS [10]. In addition, CS-related hospital admissions from LMR were also analysed.

## Results

### Results of pregnancy screening

In 2008 a blood test was offered to 190,141 pregnant women. In 2007 and 2006 the numbers were 186,141 and 185,942 respectively (table 1). HBV and syphilis tests were refused 1 to 4 times per year only. Frequency of refusal for the HIV test was higher: 376 times in 2008, 350 in 2007 and 340 in 2006 (0.2%).

**Table 1:** Results of blood tests for hiv, HBV and syphilis, 2006-2008

Year	Number refusers (%)	Number pregnancies tested	Result positive 12 weeks*	Number with confirmatory Result (%)	Result confirmed positives (%)**	Result negative Total	Prevalence estimate 1 (minimum)	Prevalence estimate 2	Prevalence estimate 3 (maximum)	95% CI (based on confirmed positives)
**HIV**										
2006	340 (0.2)	185.602	342	274 (80%)	81 (24%)	184.188	0.04	***0.05***	0.08	0.04 - 0.05
2007	350 (0.2)	185.791	327	316 (97%)	90 (27%)	183.884	0.05	***0.05***	0.05	0.04 - 0.06
2008	376 (0.2)	189.765	289	229 (79%)	68 (24%)	188.430	0.04	***0.05***	0.07	0.03 - 0.05
**HBV**										
2006	1 (<0.001)	185.941	966	927 (96%)	714 (74%)	184.621	0.38	***0.40***	0.40	0.36 - 0.41
2007	4 (<0.01)	186.137	868	848 (98%)	620 (71%)	185.015	0.33	***0.34***	0.34	0.31 - 0.36
2008	1 (<0.001)	190.140	932	896 (96%)	605 (65%)	189.771	0.32	***0.33***	0.33	0.29 - 0.34
**Syphilis**										
2006	1 (<0.001)	185.941	320	215 (67%)	142 (44%)	184.181	0.08	***0.12***	0.13	0.06 - 0.09
2007	4 (<0.01)	186.137	331	231 (70%)	181 (55%)	185.719	0.10	***0.14***	0.15	0.08 - 0.11
2008	2 (<0.001)	190.139	359	241 (67%)	197 (55%)	189.447	0.10	***0.16***	0.17	0.09 - 0.12

Excludes pregnancies not carried to term (spontaneous or through surgical abortion). HBV: 15 (2008), 22 (2007), 22 (2006); HIV: 1 (2008), 0 (2007), 1 (2006); syphilis: 1 (2008), 1 (2007), 3 (2006).

Annually between 70 to 90 women were confirmed HIV positive. These refer to the minimum numbers of HIV infections given that the HIV-confirmation was not available for everybody (3-21% unknown).

HIV prevalence was estimated to be between 0.04 and 0.08% in 2008 and was stable during the study period (table 1). False-positive HIV results occurred regularly; only 24-27% of women with a positive ELISA result also had a positive confirmation.

HBsAg-prevalence could be determined more accurately as few confirmatory results were missing (2-4%) and was estimated at 0.33% in 2008, which was a decline compared to 2006 (0.38-0.40%, χ2 test, p = 0.002). Prevalence of syphilis was between 0.10 and 0.17%, which was an increase compared to 2006 (0.08-0.13%, p = 0.02).

For hepatitis B, the confirmatory test was also positive for 65%-74% of women with an initial positive test. For syphilis these percentages ranged between 44 and 55% (table 1).

### Pregnant women with a positive blood test

**Figure 1:**
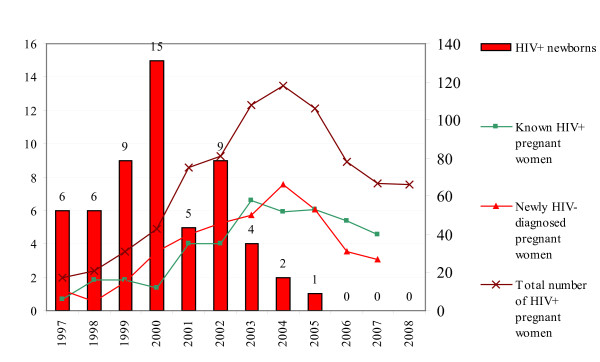
**Number HIV+ children (left y-axis) and new/known HIV+ pregnant women (right y-axis), 1997-2008 (Source: SHM, 2009)**. N.B. HIV screening of pregnant women was started in 2003 in Amsterdam.

The numbers of pregnant women with HIV in care were 66 (2008), 78 (2007) and 67 (2006) (source: SHM). In Praeventis, 68 (2008), 90 (2007) and 81 (2006) women were registered with HIV. From the SHM registration it appeared that 40% of these women were diagnosed with HIV for the first time during pregnancy (Figure 1, 2008 incomplete, not shown).

**Figure 2:**
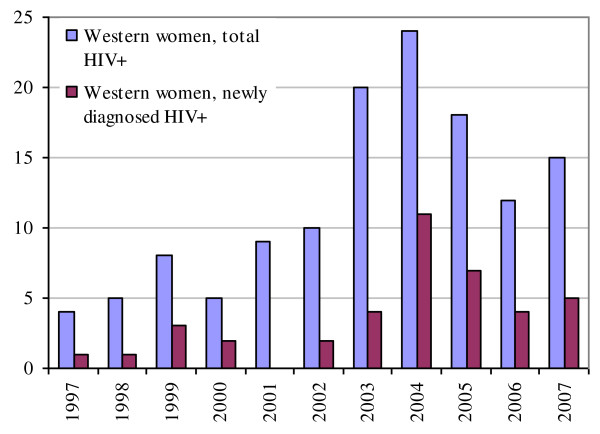
**Total newly diagnosed and known HIV positive pregnant women by Western origin (source: SHM, 2009)**.

**Figure 3:**
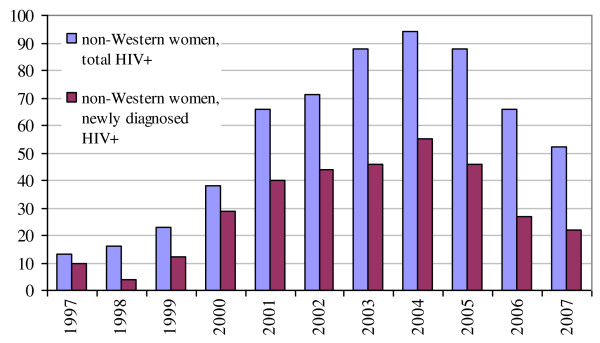
**Total newly diagnosed and known HIV positive pregnant women by non-Western origin (source: SHM, 2009)**.

The majority of women originated from countries with a generalised HIV epidemic (2007: 67%, mainly sub-Saharan Africa). Relatively more Western women were newly diagnosed after the HIV screening was introduced in 2004: 5-10 per year. In the period prior to 2004 between 0-4 were diagnosed per year (χ2, p = 0.01) (Figure 2&3).

Virtually all HBV infections involved pregnant women who are long-term carriers (2008: 99.2%). The most frequently reported regions of origin were: Asia (38%), sub-Saharan Africa (23%), Middle East (17%), Eastern and Central Europe (12%), Mid- and South America (5%) and other (5%).

### HIV, HBV and syphilis infections in newborns

The number of children born with HIV dropped from 9 in 2002 to 0 in 2008. After the introduction of screening in 2004, four children were born with HIV (Figure 1). Two mothers became pregnant before screening was introduced. One mother was HIV negative and probably became infected with HIV after the 12th week of pregnancy. The four children all had at least one parent not born in the Netherlands. No additional information was available for the fourth mother.

There were no known cases of mother-to-child transmission by HIV infected women being treated with antiretroviral therapy [7].

Five children were born with HBV between 2006-2008 (table 2). In three of these the mothers were HBeAg positive (a marker for high infectivity). Three mothers originated from China. All five children were immunised in time with the HBIg and HBV vaccine. Information regarding the mother's treatment or viral load was not registered.

**Table 2:** Results of blood tests for HIV, HBV and syphilis and the estimated number of prevented congenital infections per year, 2006-2008

	HIV	HBV	Syphilis
Positive diagnoses in pregnancies			
2005/'06*†	57	545	142
congenital infections in 2006	1	3	0
2006/'07*	85	669	143
congenital infections in 2007	0	1	5
2007/'08*	86	633	105
congenital infections in 2008	0	1	1
Average number of diagnoses in pregnancy per year (2006-2007)*	75	615	130
Through screening newly diagnosed HIV, HBV or active syphilis per year, as percentage of the total screen-positives*‡§	40%	40%	17%
Prevented children 'at risk' per year*	30	245	20
Transmission risk	20-30%	20-30%	50%
Estimated number of congenital infections prevented per year*||	5-10	50-75	± 10

*Minimum number, based on confirmed test results only

† Source: TNO-rapport 2008.

‡Sources: Stichting HIV Monitoring SHM (HIV), Osiris (HBV) and 'Research voor beleid ' - Research for policy (syphilis).

§ Estimate of the number of active syphilis infections [5]

|| Estimate based on the average transmission risk of mother to child (in the absence of treatment and/or vaccination) and the estimate of the number of new diagnoses (HIV/HBV) or active infection (syphilis) (based on women with a confirmed result)

**Figure 4:**
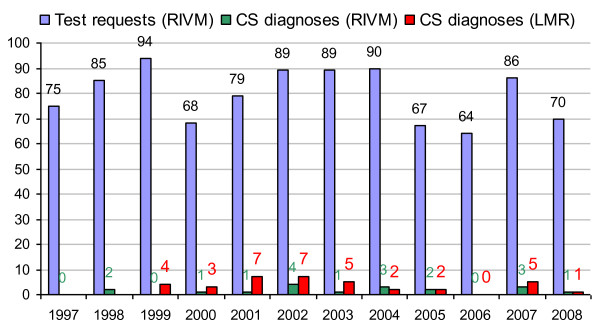
**Number of test requests for congenital syphilis for neonates and young children (< 1 year) and the number of children testing positive (IgM positive), 1997-2008 (source: RIVM, LIS)**.

Congenital syphilis was diagnosed in fewer than 5 newborns per year by the RIVM in the last ten years (Table 2, Figure 4). In all cases where newborns were infected, the mothers belonged to vulnerable groups; primarily untreated mothers (illegal immigrants or drug-users) who had withdrawn from regular pregnancy monitoring [6].

### Prevented peri-/congenital infections per year

Table 2 demonstrates the number of infections in newborns per year (January - December) in relation to the number of pregnant women in the previous six-month period (June-June, screening occurs approximately six months prior to birth).

We estimated the number of children who would have been exposed had there been no pregnancy screening or treatment. The number of peri-/congenital infections was calculated on the basis of an estimate of the number of pregnant women with confirmed HIV and HBV (both 40% of all screen positive pregnancies) discovered annually through the screening programme, the number of active syphilis infections (17%) in pregnant women with a syphilis positive result and the average risk of transmission from mother to child (HIV/HBV: 20-30%; active syphilis: 50%) (table 2) [6,11,12]. We estimated that 5-10 HIV, 50-75 HBV and 10 syphilis cases in newborns had been prevented in the Netherlands due to the annual screening.

## Discussion

Participation rates in HIV screening among pregnant women in the Netherlands are very high compared to other EU countries (99.8%), due to the opting out principle [13-15]. For 40% of the pregnant women infected with HIV the diagnosis was made for the first time during pregnancy. The number of vertical HIV transmissions has decreased significantly, after the introduction of HIV screening in 2004, to an average of 1 per year. At least 5 to 10 HIV infections were prevented in newborns annually due to the screening programme. For syphilis and HBV, the estimated numbers were 10 and 50-75.

HIV and syphilis infections, which were nevertheless diagnosed in newborns, mainly concerned children of mothers who had either not attended monitoring (on time) or had acquired the infection after the first trimester of pregnancy. Three of the five children born with HBV had mothers who were highly infectious. Perinatal transmission can still occur in pregnant women with a high viral load, despite vaccination of newborns. While HBeAg is an important marker for infectivity, ideally, for every HBsAg-positive mother a viral load determination should be made, since it is possible to have a high viral load without being HBeAg positive [16]. Treatment with antiviral agents is considered for these women, and can improve the effectiveness of the screening programme [17].

Only estimated prevalences could be derived for the three infectious diseases due to missing or difficult to interpret test results in Praeventis. There are strong indications that the number of registered syphilis infections in pregnant women is an overestimation of the number of cases of infectious syphilis. This is a result of false-positive results (through endemic treponematoses) or previously treated infections giving a positive result. At individual level there was insufficient information about which test had been carried out (VDRL or TPHA), which meant no distinction could be made between active syphilis and a previous case of syphilis. Data from a previous process evaluation [6] were used in order to calculate the number of CS infection prevented by screening. For this calculation, the midwives and gynaecologists of some of the syphilis positive women were asked whether there had been an active infection, which appeared to be case for 17%.

Although data collection for the screening of pregnant women has improved in 2008 in respect of previous years, quality assurance standards need to improve further [18]. Follow-up questions regarding missing test results regularly revealed that the confirmatory test had not been undertaken as HIV or HBV infection had already been diagnosed. This means that positive results may have been missed given that this information was not always available at an individual level. Therefore, we presented prevalences as minimum and maximum estimates. In 2009 a minimum data set was implemented in Praeventis for pregnancy screening [18]. Following this, more comprehensive background details will become available regarding newly diagnosed and known carriers.

The screening programme takes place relatively early in the pregnancy. Infections in pregnancy which are acquired after screening go undetected. In our research period infections such as these led to at least one perinatal HIV transmission. However, screening later on in the pregnancy is not effective, as maternal treatment needs to be started early (in the first trimester for syphilis). A second screening late in pregnancy is highly unlikely to be cost-effective as the incidence of HIV, HBV and syphilis infections in the Netherlands is low. This may be indicative, for example in case of a known HIV-positive partner. Advice on safe sex by the obstetric care is also important.

Prenatal screening provides a favourable cost-benefit ratio even in low-endemic countries [19,20]. Studies in Amsterdam showed that a universal screening programme is cost-effective where HIV prevalence is 5 in 10,000 (0.05%) [19,20]. The benefit of universal screening is that HIV, HBV, or syphilis positive women are detected who do not fall into a known risk profile. Due to the fact that infected women who are pregnant are being referred for treatment their clinical prognosis will improve and the risk of secondary transmission can be reduced. Universal screening is considered simpler and more acceptable than a programme only focused on risk groups.

## Conclusions

We conclude that the screening programme in the Netherlands is effective in detecting HIV, HBV and syphilis in pregnant women and in preventing transmission to the child.

Based on cost effectiveness studies in the Netherlands and other European countries, we conclude that antenatal screening is also cost effective [21]. More research is required into the background characteristics of pregnant women attending late or refusing HIV testing, as well as the desirability of re-testing high-risk women just prior to birth and those women with an unknown HIV status.

## Competing interests

The authors declare that they have no competing interests.

## Authors' contributions

All authors were involved in the development of the evaluation design and acquisition and interpretation of the data. EC, SH, and MS have been involved in drafting the manuscript. EC, YW and PO coordinated the study. CS and KB provided HIV data, DN provided syphilis data, and SH the HBV data. All authors read and approved the final manuscript.

## Pre-publication history

The pre-publication history for this paper can be accessed here:

http://www.biomedcentral.com/1471-2334/11/185/prepub
